# A Rare Case of Erythromycin-Induced Rhabdomyolysis

**DOI:** 10.7759/cureus.75882

**Published:** 2024-12-17

**Authors:** Zaid Khamis, Mohammad Aldalahmeh, Salim Barakat, Saif Abu-Baker, George Khattar

**Affiliations:** 1 Internal Medicine, Staten Island University Hospital, Staten Island, USA

**Keywords:** drug-induced rhabdomyolysis, erythromycin, macrolide induced rhabdomyolysis, rhabdomyolysis, rhabdomyolysis causing acute kidney injury

## Abstract

Rhabdomyolysis (RML) arises from the breakdown of muscle tissue, leading to the release of intracellular components into the bloodstream and potentially causing multi-organ failure. Multiple drugs have been reported to cause RML. We present here a rare instance of erythromycin-triggered RML in a patient who was not on any other potential RML-inducing medications. A 25-year-old male presented to the ED complaining of a tingling sensation and increased dyspnea with muscle aches. He took erythromycin over the counter for two days before. On presentation, diffuse muscle tenderness was found, and the vitals showed tachycardia and tachypnea. Labs showed elevated creatinine, peaking at 10.1, and elevated creatine kinase (CK) peaking at 1.2 million. He was treated in ICU with aggressive fluid resuscitation. Then he required dialysis due to fluid overload and not responding to diuretics. Extensive workup failed to find a cause for RML. This represents only the second documented instance of erythromycin-induced RML in a patient who is not concurrently using any other myotoxic medications. Before attributing the patient's condition to the erythromycin he was taking, it was essential to rule out the typical triggers of RML. Every physician must be familiar with the symptoms and prevalent triggers of RML.

## Introduction

Rhabdomyolysis (RML) denotes the dissolution of striated muscles. This condition, while relatively frequent, can pose a serious threat to life. It arises from the breakdown of muscle tissue, leading to the release of intracellular components such as potassium, phosphorus, myoglobin, creatine phosphokinase (CK), and lactate dehydrogenase (LDH) into the bloodstream, which can lead to metabolic acidosis, acute kidney injury, and subsequent multi-organ failure [1-4].

There is a wide variation in the clinical presentation of RML. Symptoms tend to reflect the primary disorder, as well as muscle injury or superimposed renal failure, ranging from a completely asymptomatic rise in serum levels of enzymes released from myocytes to severe conditions associated with intravascular volume depletion, metabolic acidosis, and electrolyte abnormalities [2]. While the classic symptom triad involves myalgia, weakness, and tea-colored urine, this triad is identified in fewer than 10% of patients, with over 50% reporting the absence of both muscle pain and weakness. [5,6].

The causes of RML have historically been categorized as genetic/acquired or traumatic/non-traumatic. While direct muscle injury continues to be the primary source of muscle damage, the onset of RML is linked to various medical conditions and pathological abnormalities. Other contributing factors encompass toxins, infections, muscle ischemia, electrolyte and metabolic imbalances, genetic disorders, exertion, prolonged immobilization, and temperature-related conditions like neuroleptic malignant syndrome (NMS) and malignant hyperthermia (MH). The final common pathway resulting in muscle injury and necrosis is direct myocyte injury or energy supply failure in the muscle cell [2,5-7].

Multiple drugs have been reported to cause RML either by their direct myotoxic effect, indirect muscle damage, or by leading to higher serum levels of co-administered myotoxic drugs. Numerous instances of macrolide-induced myopathy in patients consuming statins have been documented [8,9]. However, we present here a rare example of erythromycin-triggered RML in a patient who was not on any other potential RML-inducing medication.

## Case presentation

A 25-year-old male with a past medical history of asthma presented to the emergency department complaining of a tingling sensation throughout his body as well as increased shortness of breath and muscle aches. Two days prior to this presentation, he went to the emergency department complaining of a cough along with fever, chills, headache, and body aches for one week, for which he took erythromycin without a prescription for two days without any improvement. He was diagnosed with pneumonia and was prescribed doxycycline in urgent care. After starting the new treatment, he started to experience a tingling sensation throughout his body. He reported taking doxycycline before without any issues and that it was his first time taking erythromycin. He denied any fever or chills this time, recent exertion, drug abuse like cocaine or ethanol, or new sexual encounters. He also denied any family history of autoimmune or genetic disorders.

Upon physical examination, he had diffuse muscular tenderness with dry mucous membranes, with otherwise unremarkable examination. Vital signs were significant for tachycardia reaching up to 130 beats per minute, along with tachypnea with a respiratory rate of 24. Blood pressure was stable, and oxygen saturation was normal in the room air.

Blood work on admission revealed a creatinine on admission of 1.5 mg/dL that peaked at 10.1 mg/dL after 15 days as well as transaminitis and a significant increase in the creatine kinase (CK) levels reaching 1.2 million U/L (Table 1). Imaging including a CT chest abdomen and pelvis was negative for any acute pathologies.

**Table 1:** Laboratory values during the hospital course from admission to discharge Laboratory parameters are shown at three time points: admission, peak/significant values during hospitalization (with day of hospitalization noted in parentheses), and discharge. Notable findings include markedly elevated CK peaking at 1231700 U/L on day four, acute kidney injury with creatinine reaching 10.1 mg/dL on day 15, and significant anemia requiring transfusion with hemoglobin dropping to 6.6 g/dL on day 10. Normal ranges were not included in the original data. INR: international normalized ratio; CK: creatine kinase

Labs variable		On admission	Significant values during the stay	On discharge
Creatinine		1.5 mg/dL	10.1 mg/dL (Day 15)	2.3 mg/dL
Blood urea nitrogen		19 mg/dL	109 mg/dL (Day 15)	35 mg/dL
Aspartate aminotransferase		1001 U/L	3804 U/L (Day 4)	42 U/L
Alanine aminotransferase		190 U/L	683 U/L (Day 4)	62 U/L
Anion gap		20 mmol/L	27 mmol/L (Day 3)	13 mmol/L
CK		306340 U/L	1231700 U/L (Day 4)	1360 U/L
INR		1.06		1.03
Total bilirubin		1.0 mg/dL		<0.2 mg/dL
Albumin		4.0 g/dL		4.4 g/dL
Hemoglobin		15.6 g/dL	6.6 g/dL (Day 10)	10.7 g/dL
WBC count		7.97 K/uL	28.35 K/uL (Day 13)	5.5 K/uL
Eosinophils count		0.01 K/uL		0.13 K/uL

The patient received immediate intensive care treatment with aggressive intravenous normal saline hydration, targeting urinary output between 200-300 mL/hour. Also, both steroid therapy and broad-spectrum antibiotics were initiated to address potential infectious and inflammatory causes of RML.

Despite interventions, the patient's renal function deteriorated significantly over subsequent days. He developed severe anasarca that was refractory to both intravenous furosemide and bumetanide, necessitating the initiation of hemodialysis. Concurrent severe hypocalcemia required aggressive management with intravenous calcium gluconate supplementation and ergocalciferol administration. The patient also exhibited marked hyperphosphatemia, requiring intervention with phosphate-binding agents.

Furthermore, the clinical course was complicated by progressive anemia, requiring transfusion of two units of packed red blood cells. Although the patient maintained adequate urine output, persistent fluid overload and metabolic acidosis warranted continued dependence on hemodialysis while in the ICU.

 A comprehensive workup was done to find the etiology of RML, starting with a drug screen that was negative. Moreover, an extensive auto-immune workup including antinuclear antibody, rheumatoid factor, anti-ribonuclear protein, anti-Jo-1 antibody, PL-7 and PL-12, EJ- OJ- and SRP- myoMarker3, MI-2, fibrillarin (U3 RNP), MDA5 (P140)(CADM-140), NXP-2 (P140) Myoplus, TIF gamma (P155/140), anti PM-Scl-100, U2 snRNP, anti-U1-RNP Ab, Ku myomarker3, anti-SS-A 52 kD ab IgG, and anti-SAE 1 IgG, was negative. Also, a viral panel of respiratory viruses, hepatitis viruses, and HIV was negative. Thus, the patient's RML was attributed to erythromycin therapy.

The patient was downgraded to the general medical floor and was safely discharged from the hospital one month later with improvement in his kidney function, hypocalcemia, and anemia. He didn’t require dialysis on discharge. The patient was educated on avoiding any macrolide medication.

## Discussion

To the best of our knowledge, this is the second documented case of erythromycin-induced RML in a patient who is not concurrently using any other myotoxic medication. The previous reported case was of a 38-year-old male patient who was admitted with pneumonia and treated with erythromycin (500 mg twice daily). He subsequently developed myalgia and arthralgia only after the first day of administration; in our case, the patient’s symptoms started two days after his first dose. Both patients had elevated CK, ALT, and AST levels; in the previous case CK reached a concentration of 228456 U/L, and our patient’s CK level peaked at 1.2 million U/L. In both cases, the patients developed severe renal failure requiring hemodialysis; however, our patient was still making urine but had diuretics-resistant anasarca, while the patient in the previous case was anuric and his glomerular filtration rate (GFR) was reduced to less than 10 mL/min.

The clinical presentation of RML varies significantly, and most patients do not report muscle-related symptoms. Therefore, physicians must maintain a high index of suspicion to effectively diagnose this condition. This begins with conducting a comprehensive medical history and physical examination. The confirmation of RML hinges on clinical symptoms coupled with a serum CK level exceeding 1000 IU/L or CK surpassing five times the upper limit of normal (ULN) [4]. In the case at hand, the patient displayed diffuse muscle pain and tenderness, prompting the physician to suspect RML and initiate further assessment.

Various medications have been documented to cause RML, including antibiotics such as daptomycin, trimethoprim-sulfamethoxazole, linezolid, fluoroquinolones, and cefdinir [6,7]. Macrolides are among the most widely prescribed antibacterials, particularly for respiratory infections, and the interaction between statins and macrolides has been thought to occur due to the macrolide inhibition of the cytochrome P3A4 isoenzyme and organic anion transporter 1B, leading to heightened exposure to statins [8]. The occurrence of macrolide-induced RML is extensively documented; nevertheless, it is noteworthy that almost all previously reported instances of macrolide-induced RML involved patients concurrently taking statins [5,10-12]. Only four cases of macrolide monotherapy-induced RML were reported, among which only one patient was on erythromycin. The other three cases were for patients on clarithromycin [11-13]. However, no pathophysiologic mechanism has been described to explain this adverse effect.

Before attributing the patient's condition to erythromycin, it was essential to rule out typical triggers of RML like trauma, strenuous physical activity, infections, and electrolyte disturbances. To achieve this, a thorough patient history was obtained, followed by an extensive workup that encompassed drug screening, autoimmune assessments, and viral panels, all of which yielded negative results. By excluding other causes and taking the temporal sequence into consideration, the erythromycin adverse drug reaction here was assessed as probable according to the Naranjo adverse drug reaction probability scale [14].

One limitation of this case report is the inability to definitively establish erythromycin as the causative agent of RML, despite it being the most probable cause based on the Naranjo adverse drug reaction probability scale. The patient had been taking doxycycline, which could be a confounding factor, even though he had taken it before without any issues. Moreover, the lack of other cases with a similar presentation limits the ability to make definitive conclusions. Finally, this report is based on a single case, which makes it difficult to generalize the findings to a larger population.

## Conclusions

In conclusion, this case highlights the importance of considering drug-induced RML in patients presenting with unexplained muscle pain, weakness, and renal dysfunction, even in the absence of common risk factors such as statin use or illicit drug abuse, as effective treatment depends on accurate diagnosis. Additionally, healthcare providers should seize every opportunity to educate patients about the importance of avoiding medications without a valid prescription and should consistently inquire about the use of non-prescribed drugs, even in cases involving young, healthy individuals with no previous medical history.
